# 

**Published:** 1912-08

**Authors:** 


Vol. LXVIII AUGUST, 1912 No. 1

### BUFFALO MEDICAL JOURNAL

Established 1845 by AUSTIN FLINT M.D.

LIBRARY
AUG. 5 1912
SURGEON-GENERAL'S OFFICE

**EDITOR AND PUBLISHER:**

A. L. BENEDICT, A.M., M.D., Buffalo

**ASSOCIATE EDITORS:**

*NEW YORK STATE*

GROVER W. WENDE, M.D., Buffalo.

CHARLES W. HENNINGTON, B.S., M.D., Rochester.

CHARLES HAASE, M.D., Elmira.

FAYETTE H. PECK, M.D., Utica.

MARTIN B. TINKER, B.S., M.D., Ithaca.

*FOREIGN*

SIR WILLIAM OSLER, M.D., LL.D. Oxford, Eng.

PROF. P. K. PEL, M.D., Amsterdam, Holland.

HENRY W. HILL, LL.D., Buffalo, Associate Editor in Medical Jurisprudence.

